# Effect of rGO Coating on Interconnected Co_3_O_4_ Nanosheets and Improved Supercapacitive Behavior of Co_3_O_4_/rGO/NF Architecture

**DOI:** 10.1007/s40820-017-0141-9

**Published:** 2017-03-17

**Authors:** Tinghui Yao, Xin Guo, Shengchun Qin, Fangyuan Xia, Qun Li, Yali Li, Qiang Chen, Junshuai Li, Deyan He

**Affiliations:** 1 Key Laboratory of Special Function Materials and Structure Design of the Ministry of Education, Key Laboratory for Magnetism and Magnetic Materials of the Ministry of Education, and School of Physical Science and Technology, Lanzhou University, 222 South Tianshui Road, Lanzhou, 730000 People’s Republic of China; 2 Institute of Electromagnetics and Acoustics, Department of Electronic Science, and Fujian Provincial Key Laboratory of Plasma and Magnetic Resonance, Xiamen University, Xiamen, 361005 People’s Republic of China

**Keywords:** Supercapacitors, rGO, Co_3_O_4_ nanosheets, Strain relaxation

## Abstract

In this study, the effect of reduced graphene oxide (rGO) on interconnected Co_3_O_4_ nanosheets and the improved supercapacitive behaviors is reported. By optimizing the experimental parameters, we achieved a specific capacitance of ~1016.4 F g^−1^ for the Co_3_O_4_/rGO/NF (nickel foam) system at a current density of 1 A g^−1^. However, the Co_3_O_4_/NF structure without rGO only delivers a specific capacitance of ~520.0 F g^−1^ at the same current density. The stability test demonstrates that Co_3_O_4_/rGO/NF retains ~95.5% of the initial capacitance value even after 3000 charge–discharge cycles at a high current density of 7 A g^−1^. Further investigation reveals that capacitance improvement for the Co_3_O_4_/rGO/NF structure is mainly because of a higher specific surface area (~87.8 m^2^ g^−1^) and a more optimal mesoporous size (4–15 nm) compared to the corresponding values of 67.1 m^2^ g^−1^ and 6–25 nm, respectively, for the Co_3_O_4_/NF structure. rGO and the thinner Co_3_O_4_ nanosheets benefit from the strain relaxation during the charge and discharge processes, improving the cycling stability of Co_3_O_4_/rGO/NF.

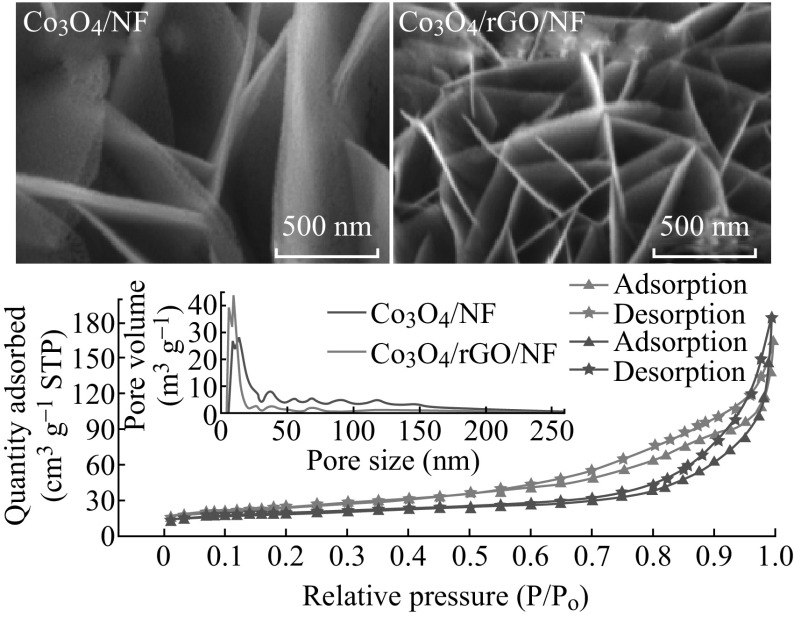

## Highlights

- Interconnected Co_3_O_4_ nanosheets anchored on rGO-coated nickel foam (NF) are facilely synthesized using a green, simple, and low-cost approach.
- Because of the high specific surface area and optimal mesopore size distribution, high specific capacitances of ~1016.4 and 767.1 F g^−1^ are achieved for Co_3_O_4_/rGO/NF at current densities of 1 and 5 A g^−1^, respectively.
- Excellent stability with ~95.5% capacity retention at a high current density of 7 A g^−1^ is achieved even after 3000 cycles.

## Introduction

In recent years, extensive efforts have been dedicated to research related to supercapacitors owing to their higher power densities, longer cycling performance than Li-ion batteries, and larger energy densities than conventional dielectric capacitors [1–3]. Supercapacitors have a huge potential in applications requiring high-density power and long cycling lifetime such as electric vehicles and portable electronics [4].

Co_3_O_4_, an important supercapacitor material, has the advantages of high theoretical capacitance (~3560 F g^−1^) [5], low cost, environmental friendliness, and high chemical stability in alkaline electrolytes. It has, thus, attracted much attention recently. Geng et al. [6] prepared porous Co_3_O_4_ nanoplates with a specific capacitance of ~231 F g^−1^ at a current density of 1 A g^−1^ using a facile reflux method. Naveen et al. [7] synthesized Co_3_O_4_/graphene nanosheets by a chemical method and a high specific capacitance of ~650 F g^−1^ at a scan rate of 5 mV s^−1^. However, there still remain some challenges in the practical applications of Co_3_O_4_ as a high capacity electrode such as poor conductivity and cycling stability, and relatively lower experimental specific capacitances than the theoretical value.

As one kind of nanostructured carbon material, reduced graphene oxide (rGO) has been extensively investigated because of its superior mechanical and electronic properties, high specific surface area, and reasonable chemical stability [8]. These properties make graphene a preferred material for use in supercapacitors and Li-ion batteries as electrode materials and/or active material supporters [9–16]. Therefore, supercapacitors combining nanostructured Co_3_O_4_ and rGO can be expected to deliver high power and energy densities and long cycling lifetime [15, 17].

In this study, interconnected Co_3_O_4_ nanosheets anchored onto rGO-coated nickel foam (NF) are facilely synthesized using a green, simple, and low-cost approach. The effect of rGO on the microstructure and improved supercapacitive behaviors of Co_3_O_4_ nanosheets are investigated here. Because of the large specific area of ~87.8 m^2^ g^−1^ and a more optimal mesopore size distribution of ~4–15 nm, the Co_3_O_4_/rGO/NF architecture delivers higher specific capacitances of ~1016.4 and 767.1 F g^−1^ at current densities of 1 and 5 A g^−1^, respectively. In comparison, the Co_3_O_4_/NF structure has a relatively smaller specific area of ~67.1 m^2^ g^−1^ and a less optimal mesopore size distribution of ~6–25 nm, resulting in lower specific capacitances of ~520.0 and 485.8 F g^−1^ at current densities of 1 and 5 A g^−1^, respectively. Moreover, Co_3_O_4_/rGO/NF has excellent stability, with ~95.5% capacity retention at a high current density of 7 A g^−1^ even after 3000 cycles. This can be attributed to the thinned Co_3_O_4_ nanosheets and the presence of rGO, improving the electrical and mechanical properties of the Co_3_O_4_/rGO/NF system. For Co_3_O_4_/NF, the capacity retention after 3000 cycles at the corresponding current density is about 84.4%.

## Experimental

### Preparation of the Co_3_O_4_/rGO/NF Architecture

**Fig. 1:**
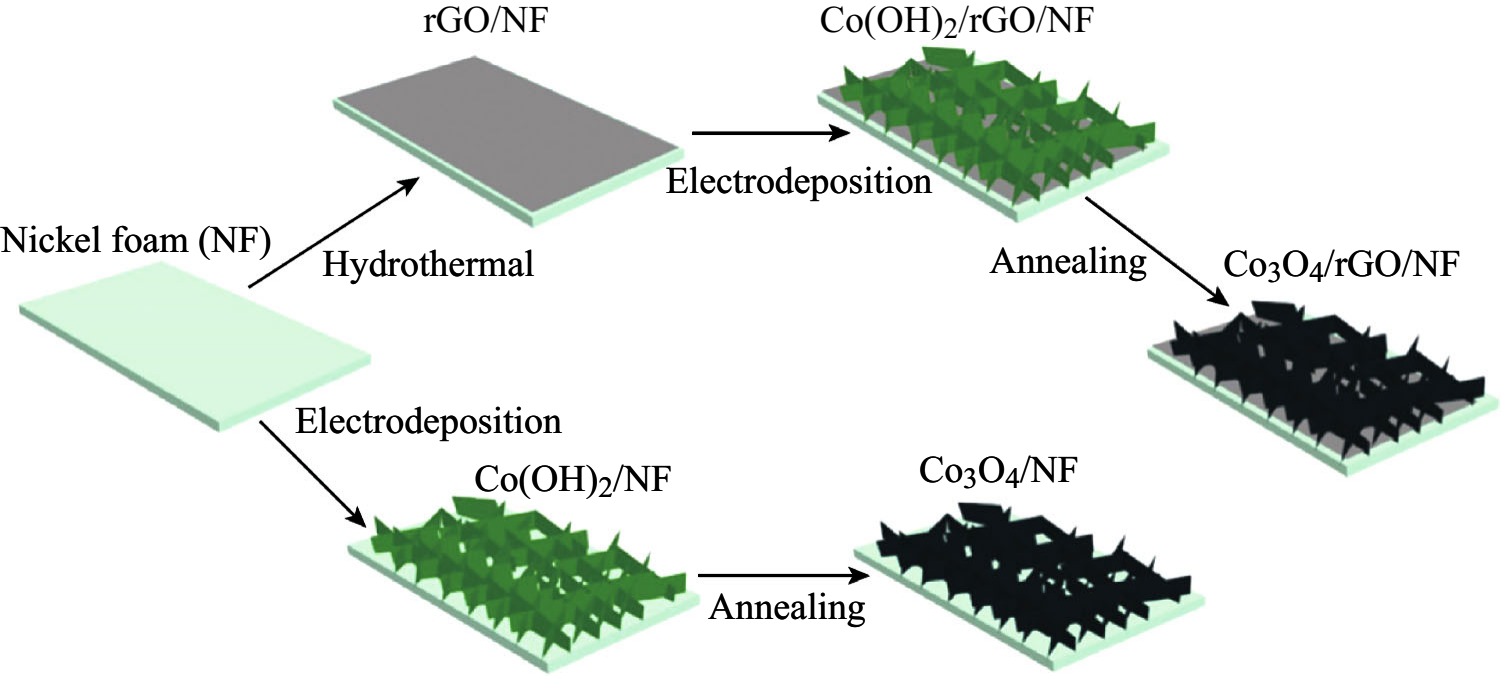
Schematic of the synthesis procedures of Co_3_O_4_/rGO/NF (*top*) and Co_3_O_4_/NF (*bottom*)

Figure 1 shows a schematic illustration of the preparation procedure of the Co_3_O_4_/rGO/NF architecture. First, the hydrothermal reduction process is used to prepare rGO-coated NF. After sequential ultrasonic cleaning in acetone, ethanol, and deionized (DI) water each for 10 min, the NFs are dried in air. GO aqueous solution (1.0 mg mL^−1^) is prepared by dispersing GO, which is prepared by the modified Hummers method [18], into DI water under ultrasonication for 30 min. Then, the GO aqueous solution (10 ml, 1.0 mg mL^−1^), ascorbic acid (L-AA, 0.02 g), and cleaned NFs of size 2 × 3 cm^2^ are placed into a beaker, which is then heated up to 95 °C and kept for 5.0 h for GO reduction and rGO coating onto the NFs. After the beaker is cooled down to room temperature, the products are taken out and dried at 50 °C for 3 h [19].

Then, Co(OH)_2_ nanosheets are electrochemically deposited at 70 °C in a three-electrode cell using rGO-coated NF as the working electrode, a platinum mesh (surface area: 2 × 2 cm^2^) as the counter electrode, and Ag/AgCl (sat. KCl) as the reference electrode. The aqueous electrolyte consists of 0.02 M Co(NO_3_)_2_·6H_2_O and 0.2 M NH_4_Cl. The electrodeposition potential is set at −3.0 V. After 500 s of electrodeposition, the resultant green foam is carefully washed using ethanol and DI water several times and finally dried in air. Then, the product is calcined at 250 °C for 2 h in a quartz tube to change Co(OH)_2_ into interconnected Co_3_O_4_ nanosheets [20]. A reference structure of interconnected Co_3_O_4_ nanosheets anchored onto NF is prepared following a similar process but without the rGO coating procedure, to determine the role of rGO.

### Characterization

The resultant products are characterized by scanning electron microscopy (SEM, MIR A3, TESCAN), high-resolution transmission electron microscopy (HRTEM, FEI Tecnai G^2^ F30), micro-Raman spectroscopy (Jobin–Yvon Horiba HR800 with an excitation wavelength of 532 nm), X-ray powder diffraction (XRD, X’Pert Philips), and Brunauer–Emmett–Teller measurement (BET, ASAP 2020 Micromeritics).

Cyclic voltammetry (CV), galvanostatic charge/discharge (GCD), and electrochemical impedance spectroscopy (EIS) tests are conducted using an electrochemical workstation having a three-electrode configuration. Co_3_O_4_/rGO/NF or Co_3_O_4_/NF is employed as the working electrode using a 1 M KOH electrolyte. The applied potential window of CV measurements ranges from 0.0 to 0.6 V. GCD is performed at a constant current over a fixed potential range of 0.0–0.5 V. The capacitance retention test is performed from 0.0 to 0.5 V at a constant current density of 7 A g^−1^ for 3000 cycles. EIS measurement is performed under an AC voltage with a 5 mV amplitude over a frequency range of 0.01-100 kHz. The specific capacitance *C*_s_ (F g^−1^) is calculated from the GCD data using *C*_s_ = (*I*△*t*)/(*m*△*V*), where *I* is the charge–discharge current, Δ*t* is the discharge time, *m* is the mass of Co_3_O_4_, and Δ*V* is the potential change.

## Results and Discussion

**Fig. 2:**
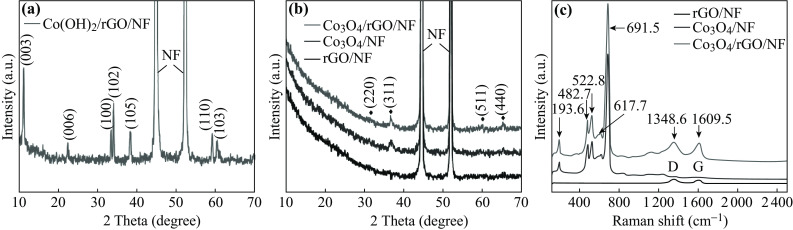
XRD patterns of **a** Co(OH)_2_/rGO/NF and **b** rGO/NF, Co_3_O_4_/NF, and Co_3_O_4_/rGO/NF. **c** Micro-Raman spectra of rGO/NF, Co_3_O_4_/NF, and Co_3_O_4_/rGO/NF composites

Figure 2a shows the XRD pattern of the Co(OH)_2_/rGO/NF structure. The peaks presented in the spectrum match well with those in the standard crystallographic spectrum of layered *α*-Co(OH)_2_ (JCPDS 46-0605) [21], except for the peaks at 44.5° and 51.8° attributed to the NF substrate (JCPDS 87-0712). Figure 2b exhibits the XRD spectra of the products of Co(OH)_2_/rGO/NF and Co(OH)_2_/NF after calcination at 250 °C for 2 h, and the XRD pattern of the rGO/NF structure serves as a reference. It is evident that except for the diffraction peaks from NF, all the other peaks are consistent with the (220), (311), (511), and (440) planes in the standard Co_3_O_4_ pattern (JCPDS 42-1467) [22]. It is worth noting that there are no obvious XRD signals of rGO, possibly because of the low mass loading or destruction of regular stacks of rGO [23]. Figure 2c shows the micro-Raman spectra of rGO/NF, Co_3_O_4_/NF, and Co_3_O_4_/rGO/NF composites. Two bands located around 1348.6 and 1609.5 cm^−1^ are assigned to the D and G band of rGO, respectively, indicating that GO has been successfully reduced [24]. For Co_3_O_4_-containing samples, five peaks of the crystalline Co_3_O_4_ phase corresponding to the A_1g_ (691.5 cm^−1^), F_2g_ (617.7 cm^−1^), F_2g_ (522.8 cm^−1^), E_g_ (482.7 cm^−1^), and F_2g_ (193.6 cm^−1^) modes are evident [25], and no obvious characteristic peaks related to Co(OH)_2_ can be observed. Accordingly, both XRD and Raman spectra indicate that Co(OH)_2_ is completely changed to Co_3_O_4_ after the aforementioned thermal treatment.

**Fig. 3:**
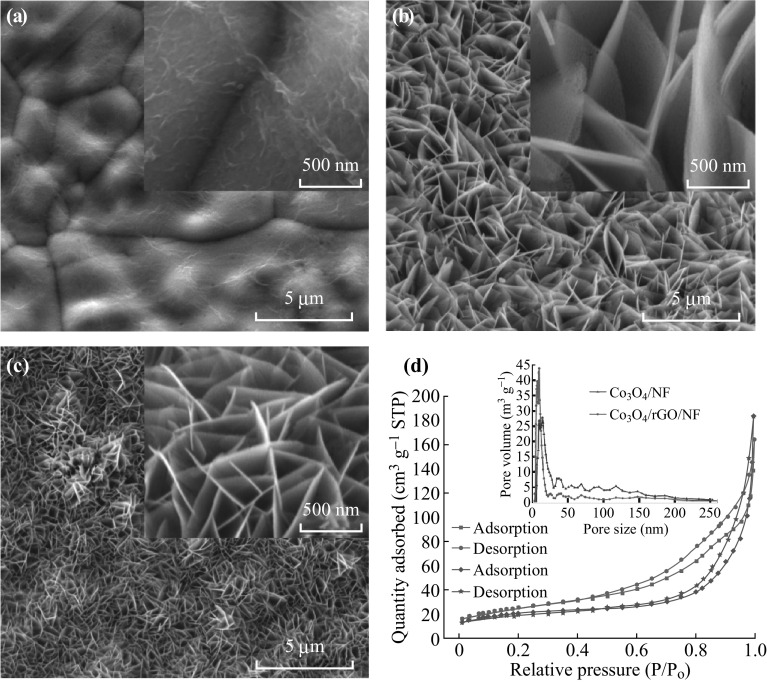
Top-view SEM images of **a** rGO/NF, **b** Co_3_O_4_/NF, and **c** Co_3_O_4_/rGO/NF. **d** N_2_ adsorption–desorption isotherms of Co_3_O_4_/rGO/NF and Co_3_O_4_/NF. The *inset* in **d** shows the pore size distribution of both samples

SEM images of rGO/NF, Co_3_O_4_/NF, and Co_3_O_4_/rGO/NF are shown in Fig. 3. It is obvious that the rGO coating onto NF is relatively uniform during the hydrothermal reducing process (see Fig. 3a). Figure 3b, c exhibits the top-view SEM images of Co_3_O_4_/NF and Co_3_O_4_/rGO/NF, respectively. Interconnected Co_3_O_4_ nanosheets can be synthesized on both NF and rGO-coated NF. This indicates that the growth of Co_3_O_4_ nanosheets is dependent more on the growth condition [26] rather than on the substrates. However, compared to the Co_3_O_4_ nanosheets on NF, the nanosheets on rGO-coated NF have a higher density and lower dimension because of the roughened NF surface due to rGO. Moreover, the corresponding high-magnification SEM images shown in the inset in Fig. 3b, c clearly show that the Co_3_O_4_ nanosheets on rGO-coated NF are thinner than the ones on NF. Accordingly, compared to Co_3_O_4_/NF, a higher specific surface area can be expected for Co_3_O_4_/rGO/NF. BET measurement is conducted to examine this hypothesis. Figure 3d shows the N_2_ adsorption–desorption isotherms of Co_3_O_4_/rGO/NF and Co_3_O_4_/NF. Two distinct hysteresis loops can be observed with a type IV sorption behavior, indicating the presence of a typical mesoporous microstructure for Co_3_O_4_/rGO/NF and Co_3_O_4_/NF. The pore size distribution of Co_3_O_4_ nanosheets on rGO (see the inset of Fig. 3d) indicates that the size of most pores is ~4–15 nm. In contrast, the size of most pores of the Co_3_O_4_ nanosheets on NF is 6–25 nm. The BET specific areas for Co_3_O_4_ nanosheets on rGO and on NF are ~87.8 and 67.1 m^2^ g^−1^, respectively. Accordingly, a higher specific capacitance can be expected for the Co_3_O_4_/rGO/NF structure.

**Fig. 4:**
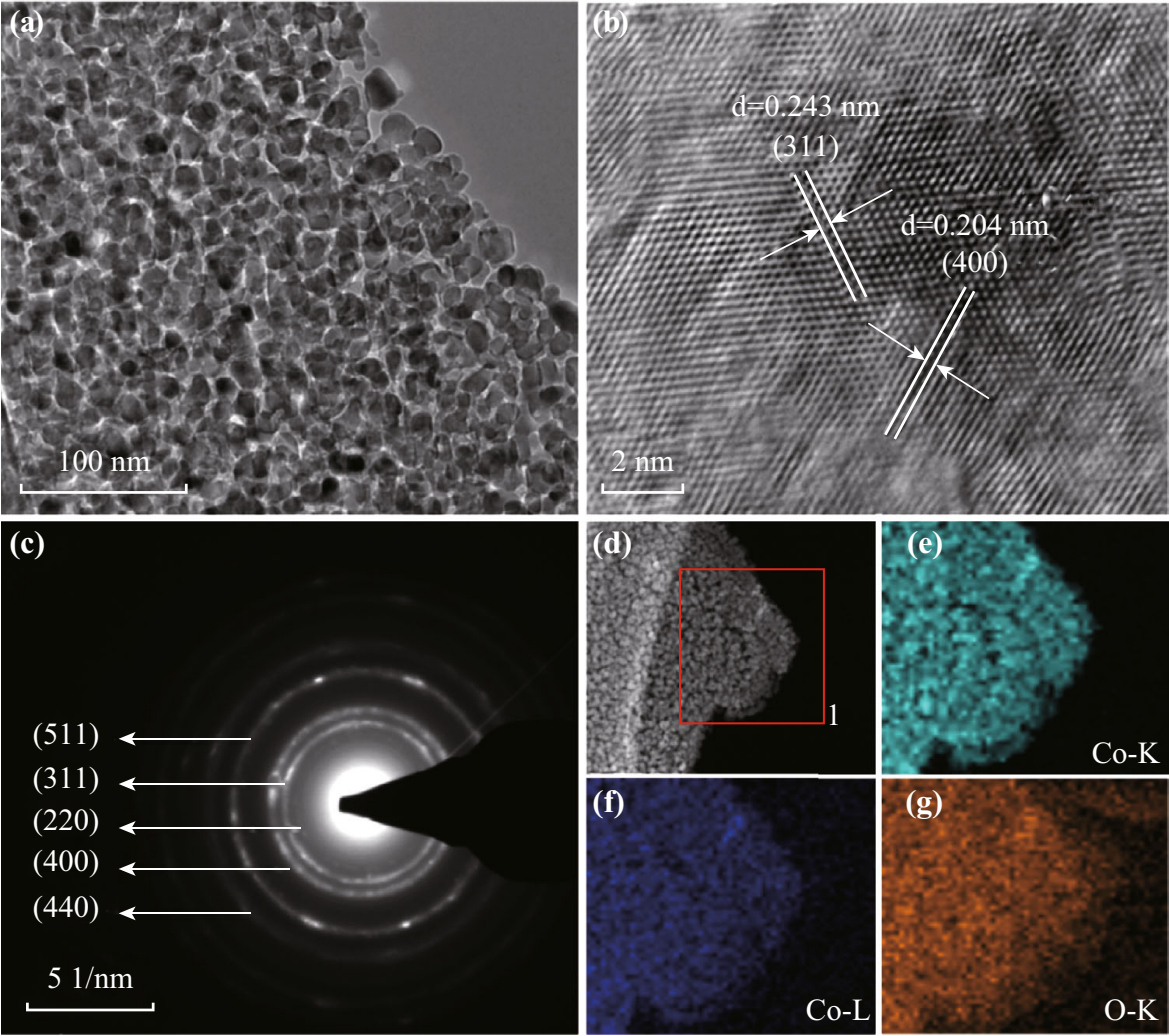
**a** Low- and **b** high-magnification TEM images, **c** SAED patterns of a Co_3_O_4_ nanosheet taken from Co_3_O_4_/rGO/NF. **d** STEM image, and elemental mapping images of **e** and **f** Co and **g** O

To reveal further details of the microstructure of the Co_3_O_4_ nanosheets in Co_3_O_4_/rGO/NF, HRTEM characterization is performed. As demonstrated in Fig. 4a, the Co_3_O_4_ nanosheets are composed of small Co_3_O_4_ nanoparticles of size ~5–15 nm. The lattice fringes as exhibited in Fig. 4b show an interplanar spacing of 0.243 and 0.204 nm, attributed to the (311) and (400) planes of cubic Co_3_O_4_ [27, 28]. The polycrystalline structure of the obtained Co_3_O_4_ nanosheets is further confirmed by the selected area electron diffraction (SAED) pattern (see Fig. 4c). The homocentric diffraction rings (from the inside to the outside) can be assigned to the (220), (311), (400), (511), and (440) planes of Co_3_O_4_ [29]. The elemental distribution in the Co_3_O_4_ nanosheets is also characterized by scanning transmission electron microscopy (STEM) (see Fig. 4d) and X-ray elemental mapping images (see Fig. 4e–g). The elemental mapping further indicates that Co_3_O_4_ nanosheets were synthesized successfully.

**Fig. 5:**
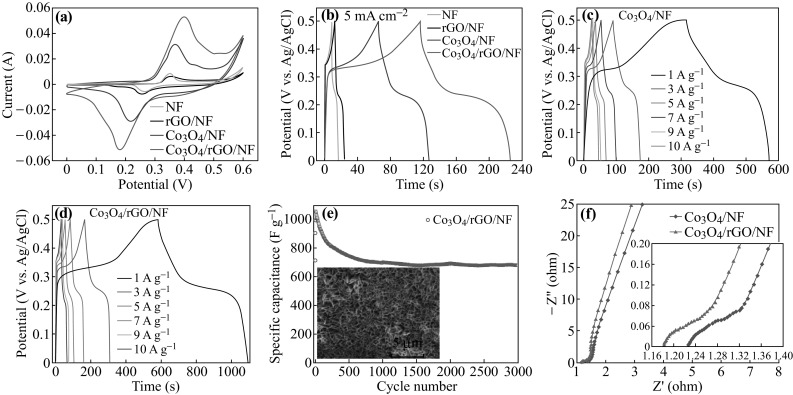
**a** CV curves of NF, rGO/NF, Co_3_O_4_/NF, and Co_3_O_4_/rGO/NF composites at 3 mV s^−1^. **b** Charge–discharge curves of NF, rGO/NF, Co_3_O_4_/NF, and Co_3_O_4_/rGO/NF composites at 5 mA cm^−2^. Charge–discharge curves of **c** Co_3_O_4_/NF and **d** Co_3_O_4_/rGO/NF at different current densities. **e** Cycling performance of Co_3_O_4_/rGO/NF at a current density of 7 A g^−1^ (the *inset* is a top-view SEM image of Co_3_O_4_/rGO/NF after the 3000 cycles shown in **e**. **f** Nyquist plots of Co_3_O_4_/rGO/NF and Co_3_O_4_/NF

Figure 5a presents the CV data for NF, rGO/NF, Co_3_O_4_/NF, and Co_3_O_4_/rGO/NF under a scanning rate of 3 mV s^−1^ and a potential range of 0.0–0.6 V. It is remarkable that the CV area of Co_3_O_4_/rGO/NF is evidently larger than that of Co_3_O_4_/NF owing to the high specific area of Co_3_O_4_/rGO/NF as mentioned above. The redox peaks for the Co_3_O_4_-containing samples originate from the conversion of different cobalt oxidation states. Here, it is worth noting that two redox peaks also emerge for NF and rGO/NF, which are attributed to the oxidized surface of NF during heat treatment of Co(OH)_2_. However, the contribution of the surface nickel oxide and rGO is not taken into consideration in the following study because of their negligible capacities compared to the Co_3_O_4_-containing samples. This is verified by the calculated areal specific capacitances of 84.0, 120.7, 627.3, and 1107.5 mF cm^−2^ for NF, rGO/NF, Co_3_O_4_/NF, and Co_3_O_4_/rGO/NF, respectively, as exhibited in Fig. 5b. The GCD results of Co_3_O_4_/NF and Co_3_O_4_/rGO/NF are exhibited in Fig. 5c, d with current densities of 1, 3, 5, 7, 9, and 10 A g^−1^ and a potential window of 0.0–0.5 V. For each charge/discharge current density, Co_3_O_4_/rGO/NF exhibits a larger specific capacitance than Co_3_O_4_/NF. The specific capacitance of Co_3_O_4_/rGO/NF is ~1016.4, 872.3, 767.1, 707.2, 657.3, and 633.0 F g^−1^ at the corresponding current density of 1–10 A g^−1^. As a distinct comparison, the Co_3_O_4_/NF electrode exhibits specific capacitances of 520.0, 513.7, 485.8, 456.8, 444.5, and 435.5 F g^−1^ at the corresponding current densities. Moreover, compared to other previously reported Co_3_O_4_/rGO composites with different Co_3_O_4_ microstructures [7, 16, 30], the Co_3_O_4_/rGO/NF electrode reported here also delivers superior specific capacitances especially at higher current densities. Here, it is worth noting that the specific capacitance decreases as the charge/discharge current density increases for both Co_3_O_4_/rGO/NF and Co_3_O_4_/NF. This is because of the insufficient supply of active material [31] and severe polarization [32] at higher current densities. Hence, how to improve the supercapacitive behaviors from these two aspects will be the main consideration in a future study.

To evaluate the cycling performance, the electrochemical stability of Co_3_O_4_/rGO/NF and Co_3_O_4_/rGO is tested using the GCD technique. The cycling performance of Co_3_O_4_/rGO/NF at a current density of 7 A g^−1^ is recorded over the potential range of 0.0–0.5 V (see Fig. 5e). For the Co_3_O_4_/rGO/NF structure, ~95.5% of the initial specific capacitance can be retained after 3000 cycles even at such a high current density compared to other related reports [7, 33]. However, ~84.4% of the initial specific capacitance is retained after the same cycling period for Co_3_O_4_/rGO. To understand this excellent cycling stability, SEM characterization is performed on the Co_3_O_4_/rGO/NF sample after 3000 cycles and is shown in the inset of Fig. 5e. It is obvious that even after 3000 cycles, the microstructure is maintained very well, showing no evident change compared to the microstructures before cycling (see Fig. 3c). It is noted that during the first cycle, there is a significant increase in the specific capacitance. Previous reports mentioned this phenomenon as an activation process [34]. However, such a strong ‘activation’ has been rarely reported and needs to be further investigated to reveal the underlying mechanism.

EIS is used to further understand the electrical properties of the related material/structural system. Figure 5f shows the Nyquist plots of Co_3_O_4_/rGO/NF and Co_3_O_4_/NF. The linear portion of the Nyquist plots in the low-frequency region corresponds to the Warburg impedance, which is related to electrolyte diffusion into the pores in the electrodes. If the impedance plot increases sharply and tends to become a vertical line, it indicates a pure capacitive behavior. In the high-frequency region, the Z’-intercept represents the equivalent series resistance (ESR) including the ionic resistance of the electrolyte, intrinsic resistance of the substrate, and contact resistance at the interface of the active material and current collector [35]. The ESR of Co_3_O_4_/rGO/NF and Co_3_O_4_/NF is ~1.18 and 1.22 Ω, respectively (see the inset in Fig. 5f), indicating a lower solution resistance and Faradaic resistance for the Co_3_O_4_/rGO/NF architecture. In this study, the improved performance of Co_3_O_4_/rGO/NF is mainly attributed to the introduction of rGO, which not only provides effective electrolyte accessible channels, thus shortening the ion diffusion distance, but also improves the specific surface area of Co_3_O_4_ and optimizes the mesopore size distribution for facilitating an enhancement in the capacitance performance.

## Conclusions

This work reports the effect of rGO on the interconnected Co_3_O_4_ nanosheets and the improved supercapacitive behaviors. It is found that rGO can help to optimize the microstructures of the interconnected Co_3_O_4_ nanosheets, including an increased specific surface area and a more optimal mesopore size distribution, which result in the specific capacitance of the Co_3_O_4_/rGO/NF architecture being higher that that of the Co_3_O_4_/NF structure. Further, the Co_3_O_4_/rGO/NF structure possesses excellent cycling stability owing to the improved mechanical and electrical properties associated with the thinned Co_3_O_4_ nanosheets and incorporation of rGO.
